# Correlation between peripheral blood neutrophil-lymphocyte ratio and CD34 expression in prostate cancer

**DOI:** 10.1186/s12885-020-07382-3

**Published:** 2020-09-22

**Authors:** Yiyang Wang, Xiaofei Dong, Zhaokui Qu, Kang Peng, Xiaolei Sun, Renfu Chen

**Affiliations:** 1 Department of Urology, Yancheng Third People’s Hospital, Yancheng, 224000 Jiangsu China; 2 Department of Urology, Xuzhou Medical College, Xuzhou, 221000 Jiangsu China; 3 Department of Urology, Xuzhou City Hospital of traditional Chinese Medicine, Xuzhou, 221000 Jiangsu China; 4 Department of Urology, Affiliated Hospital of Xuzhou Medical College, 99 Huaihai Road, Xuzhou, 221000 Jiangsu Province People’s Republic of China

**Keywords:** Neutrophil-to-lymphocyte ratio, CD34, Prostate cancer, Microvessel density

## Abstract

**Backgrounds:**

The association of neutrophil-lymphocyte ratio (NLR) and CD34 expression level with PSA level, Gleason score, and clinical stage was investigated in patients with prostate cancer. The correlation between NLR and CD34 expression was also investigated to provide evidence supporting the use of NLR for predicting the prognosis of prostate cancer patients.

**Methods:**

Clinical data of 75 patients diagnosed with prostate cancer by prostate aspiration biopsy were retrospectively analyzed. The correlation between NLR, CD34 expression, and clinicopathological characteristics was analyzed using the χ2 test and one-way analysis of variance. The correlation between NLR and CD34 was determined using the Pearson coefficient. Disease free survival was estimated by Kaplan-Meier analysis.

**Results:**

Both NLR and CD34 expression were significantly positively correlated with PSA, Gleason score, and clinical stage (*P* < 0.05 both). Patients in the NLR^High^/CD34^High^ group were characterized by high PSA level and Gleason score and late clinical stage. NLR was positively correlated with CD34 expression (*r* = 0.529, *P* < 0.001).

**Conclusions:**

Pretreatment NLR was a valuable marker of prognosis in prostate cancer. NLR is positively correlated with CD34 expression.

## Backgrounds

Prostate cancer is a common malignant tumor affecting the life of middle-aged and elderly men. It is the second most common malignant tumor causing male death in Western countries [1]. Although the incidence of prostate cancer in China is lower than that in Western countries, it has shown an increasing trend in recent years because of the aging population and improved life expectancy. Currently, the prediction of prognosis in prostate cancer is based on prostate specific antigen (PSA) level, Gleason score, and clinical stage. Recent studies suggest that neutrophil-lymphocyte ratio (NLR) is closely related to the poor prognosis of some cancers [2–5]. NLR is a systemic inflammation indicator that can be conveniently measured [6]. However, there is little evidence supporting the value of NLR for the prediction of prognosis in prostate cancer.

Intense tumor neovascularization is closely associated with tumor growth and metastasis. Angiogenesis is a key step involved in solid tumor growth. Without neovascularization, tumor volume is generally below l–2 mL, and the tumor may remain dormant or even degenerate [7, 8]. Angiogenesis is thereby a crucial factor affecting the prognosis of cancer patients. We speculate tumor angiogenesis may provide evidence for the value of NLR to predict the prognosis in prostate cancer, and to our knowledge, no studies have assessed the idea of our paper. In 1995, Weidner et al. firstly put forward the concept of tumor microvessel density (MVD) and proposed a measurement method. Immunohistochemical staining of tumor tissues enables counting microvessels under high-power field microscopy [9]. Among microvascular immunohistochemical markers, CD34 has the best sensitivity and stability with a high positive rate and expression level. CD34 is expressed in the small blood vessels of tumor tissues [10]. Moreover, the expression level of CD34 in the endothelium of newly-formed blood vessels is higher than that in old blood vessels, suggesting that CD34 is involved in tumor neovascularization [11]. Bettencourt et al. found that neovascularity as measured by the CD34 antigen may be a prognostic marker of recurrence for prostate cancer patients after radical prostatectomy [12]. We therefore selected CD34 as an indicator of MVD in the present study.

We retrospectively analyzed the clinical data of prostate cancer patients admitted to the Department of Urology of Xuzhou Medical University between September 2015 and July 2018. Pre-treatment NLR values and CD34 expression levels in tumor tissue samples were analyzed to explore their association with PSA, Gleason score, and tumor stage. The correlation between NLR and CD34 was also investigated. This study is expected to provide experimental evidence for the use of NLR in the evaluation of prognosis in prostate cancer.

## Methods

### Patients and follow-up

Seventy-five patients who underwent prostate aspiration biopsy and were pathologically diagnosed with prostate cancer between September 2015 and July 2018 in the Affiliated Hospital of the Xuzhou Medical University were included in this study. Patients were eligible if they did not receive radiochemotherapy, endocrine therapy, or surgery before biopsy. Patients with acute inflammation, hematological diseases, and other malignant tumors were excluded.

Patient information including name, age, pretreatment test results such as routine blood tests (neutrophil and lymphocyte count), total PSA, and pathological results such as Gleason score and clinical stage were collected from electronic medical records.

Patients’ post-treatment disease progression data were obtained by telephone follow-up or review of medical records. The follow-up deadline was December 2018. Disease progression was defined as biochemical recurrence after radical prostatectomy (2 consecutive PSA values ≥0.2 ng/mL after radical surgery), or progression to castration-resistant prostate cancer after endocrine therapy (serum testosterone reaching castration levels, PSA increasing in 3 consecutive times 1 week apart and a 50% or higher increase compared with the lowest value), or development of new metastases.

### NLR measurement

**Fig. 1:**
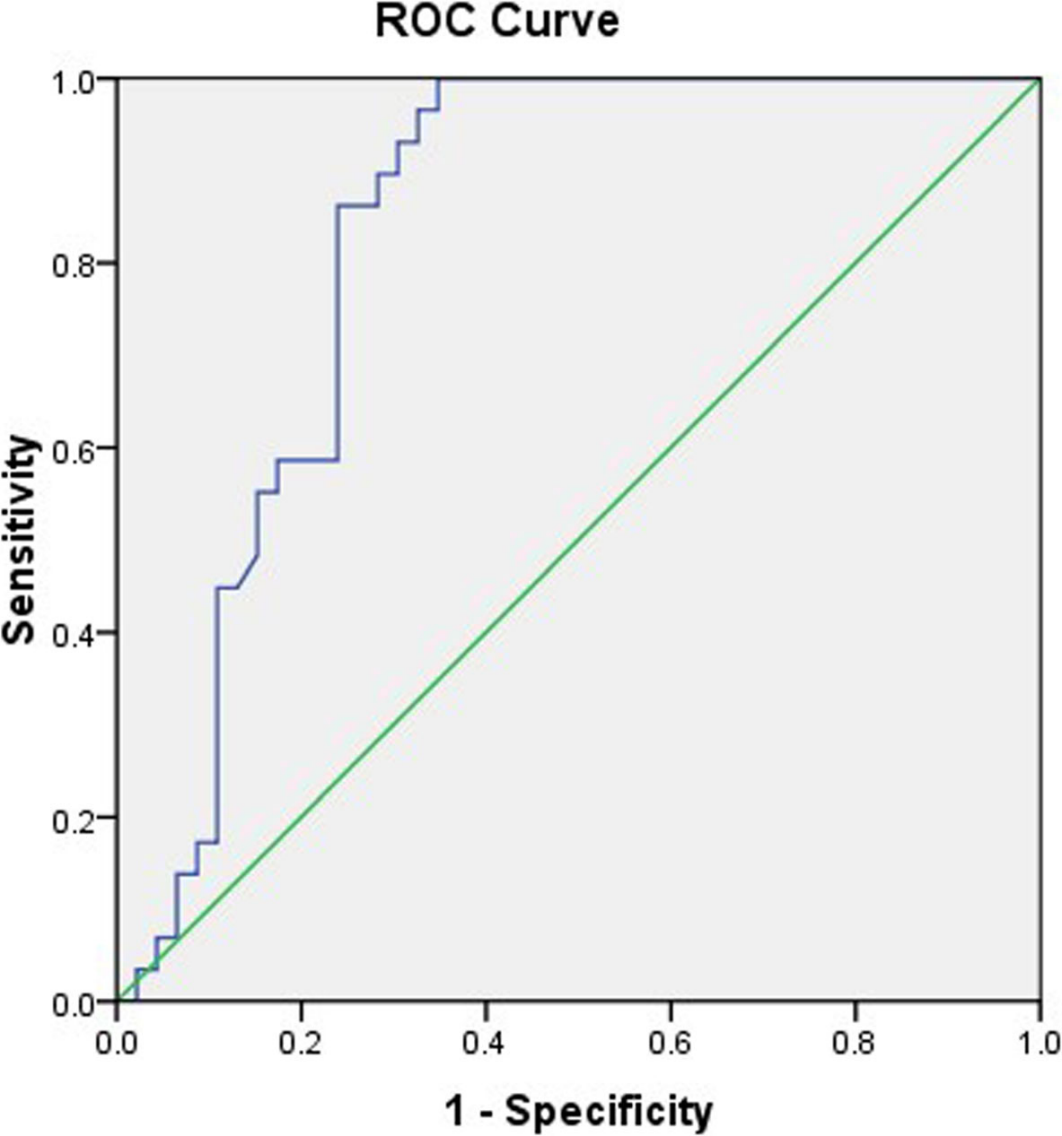
ROC curve of pretreatment NLR values and disease progression. The optimal cutoff value was 3.3 based on the ROC of NLR value and disease progression (*P* = 0.008)

NLR was calculated as the absolute neutrophil count divided by the absolute lymphocyte count (× 10^9^/L) by routine blood tests. According to the receiver operator characteristic (ROC) curve of pretreatment NLR values and disease progression in the patients and taking into account sensitivity and specificity, the corresponding sensitivity and specificity of the NLR value were highest when the Youden index (Youden index = sensitivity + specificity − 1) was the largest with the best cutoff of 3.3 (*P* = 0.008) (Fig. 1, Table 1).

**Table 1:** The best cutoff of the NLR value

	AUC	95% CI		Cutoff	Youden index	Sensitivity	Specificity	*P*-value
		Lower	Upper					
NLR	0.829	0.735	0.924	3.3	0.652	77%	91%	0.000

*NLR* neutrophil-to-lymphocyte ratio, *AUC* area under curve, *CI* confidence interval

### Immunohistochemistry

Immunohistochemistry (Streptavidin/Peroxidase, SP method) was used to detect the expression of CD34 in prostate cancer tissue samples of the 75 patients, and MVD was calculated. Prostate tissues were fixed in 10% formaldehyde in PBS, embedded in paraffin, and cut into 5-μm sections. Sections were deparaffinized in xylene and rehydrated in different concentrations of ethanol. The sections were then immersed in 0.3% hydrogen peroxide for 30 min to block endogenous peroxidase activity. Primary antibodies, which were obtained from Maxin Biological Technology (catalogue number. Kit − 0004, Fuzhou, China; monoclonal mouse anti-human CD34 antibody), were added to slides and incubated at 4 °C overnight. Following primary antibody incubation, sections were stained using the labeled anti-Rabbit/Mouse polymer (catalogue number. 0017, Long Island Antibody, Shanghai, China) for 60 min. Proteins were visualized using a liquid diaminobenzidine detection kit (Long Island Antibody, Shanghai, China). Sections were counterstained with hematoxylin for 15 min, dehydrated in different grades of alcohol, and cleared in xylene.

**Fig. 2:**
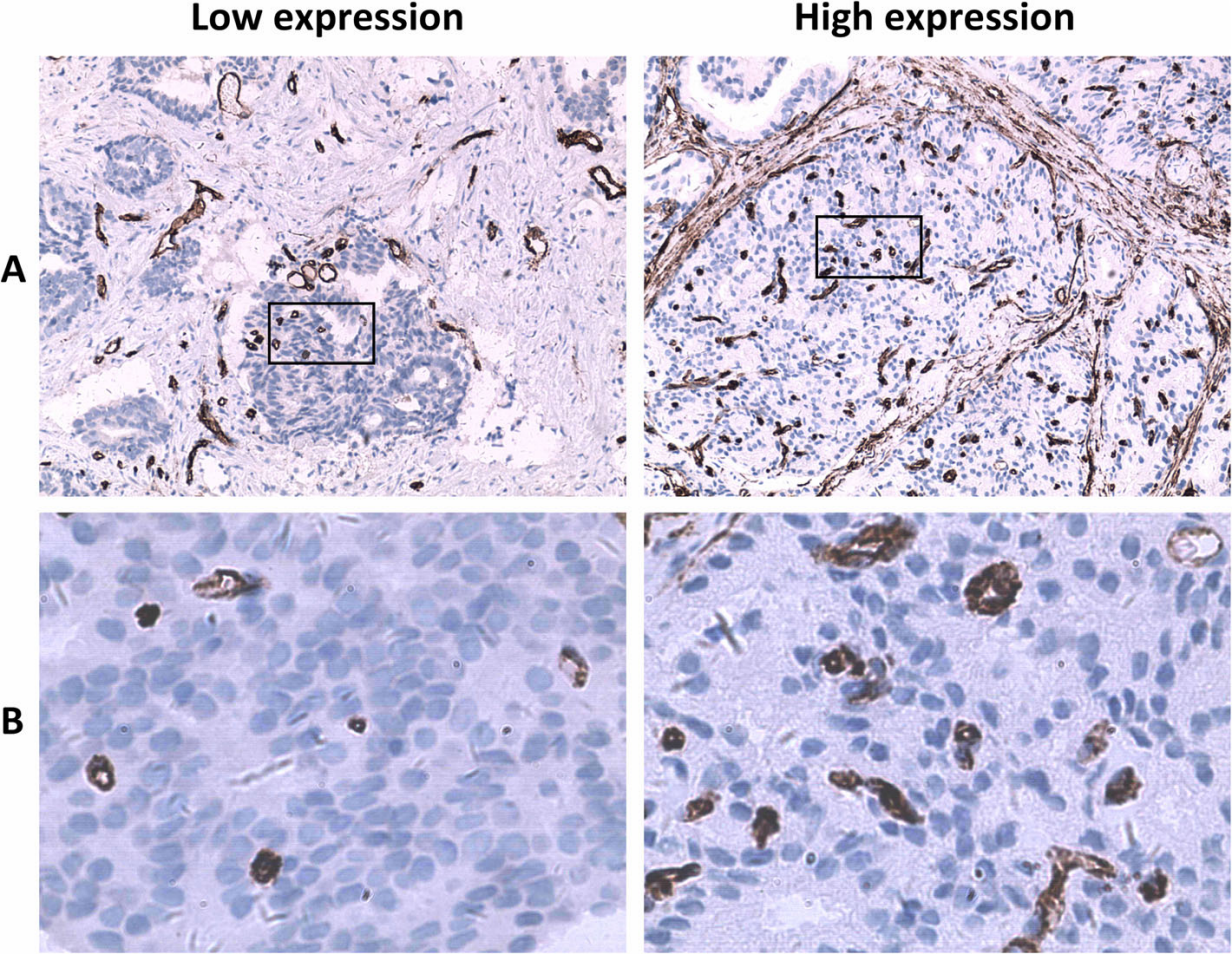
Immunohistochemistry showed expression of CD34 in prostate cancer. These images were taken at 100× magnification (**a**) and 400× magnification (**b**)

**Fig. 3:**
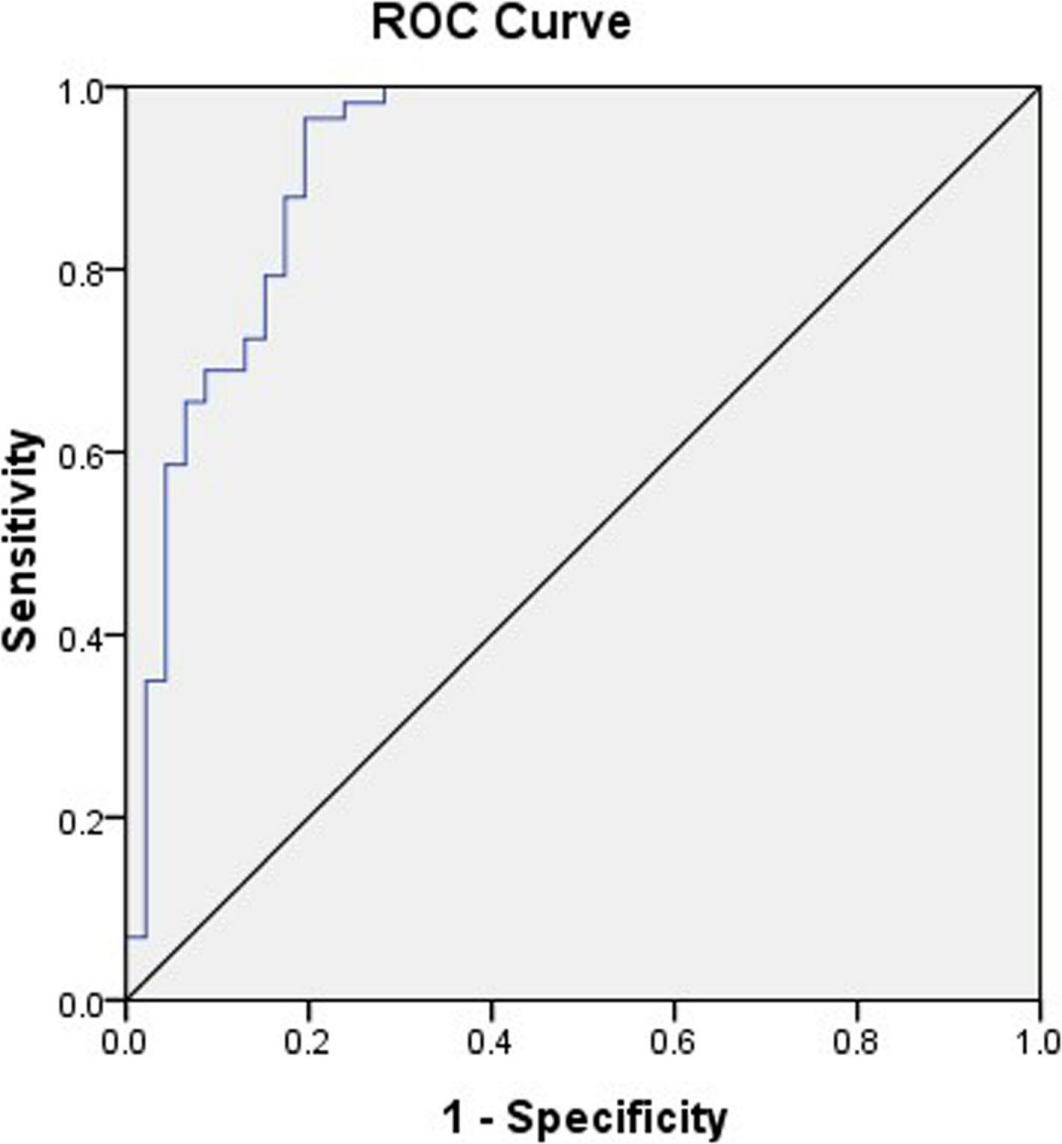
ROC curve of CD34-labeled microvessel count and disease progression. The optimal cutoff value was 26 based on the ROC of microvessel count and disease progression (*P* = 0.014)

CD34 in tumor tissues was labeled by immunohistochemistry and microvessels were brown-color stained (Fig. 2). Under the microscope, a single brown-stained endothelial cell or a cell mass was counted as a blood vessel regardless of whether the lumen was formed or not, as long as it could be clearly differentiated from tumor tissues. Vessels with a lumen diameter greater than that of eight red blood cells or those with a muscle layer were not counted. Each immunohistochemical section was first observed under a low-power field (100×) to determine the detection area with the highest MVD, and then the section was observed under a high-power field (400×) to count the number of CD34-positive microvessels in 10 non-repetitive fields. The mean value of the 10 fields was the MVD value. The optimal cutoff value was 26 based on the ROC of microvessel count and disease progression (*P* = 0.014) (Fig. 3, Table 2).

**Table 2:** The best cutoff of the CD34 value

	AUC	95% CI		Cutoff	Youden index	Sensitivity	Specificity	*P*-value
		Lower	Upper					
CD34	0.931	0.876	0.986	26	0.757	82%	93%	0.000

*AUC* area under curve, *CI* confidence interval

### Statistical analysis

Statistical analysis was performed using SPSS 16.0 statistical software. Data were expressed as the mean ± standard deviation $\left(\overline{x}\pm \mathrm{S}\right)$. The independent sample t-test was used to compare means between two groups. The relationship between NLR or CD34 and clinicopathological features was analyzed by the χ2 test. The relationship between NLR/CD34 and clinicopathological features was analyzed by analysis of variance (ANOVA). Pearson correlation analysis was used to evaluate the correlation between NLR and CD34. The Kaplan-Meier method was used to analyze disease-free survival, and the log-rank test was used to determine significance. *P* < 0.05 was considered to be statistically significant. Figures were plotted using GraphPad Prism 5 software.

## Results

### Clinicopathological charateristics of the patients

There were 75 patients who met the inclusion criteria, and the average age was 64 years old (44–81 years old). PSA levels ranged from 2.3 to 203.5 ng/mL, with an average of 24.1 ng/mL. Regarding the Gleason score, there were 14 cases with ≤6 points, accounting for 18.7%, and 28 cases with 7 points and 33 cases with 8–10 points, accounting for 37.3 and 44.0%, respectively. There were 33 cases of stage T1–2 and 42 cases of stage T3–4, accounting for 44.0 and 56.0%, respectively. In terms of metastasis, 46 cases (61.3%) had no lymph node metastasis and 29 cases (38.7%) had lymph node metastasis, and there were 43 and 32 cases with and without distant metastasis, accounting for 57.3 and 42.7%, respectively. There were 26 stage II cases (34.7%) and 49 stage III-IV cases (65.3%). There were 32 cases (42.7%) with NLR ≤3.3 and 43 cases (57.3%) with NLR > 3.3 NLR. Representative examples of CD34 positively stained vessels in prostate cancer tissues are shown in Fig. 2, respectively. MVD measured by CD34 staining showed 36 cases (48.0%) with MVD ≤26 and 39 cases (52.0%) with MVD > 26. Fourty-six patients (61.3%) progressed and 29 patients (38.7%) had no disease progression.

### Association between NLR and CD34 respectively with clinicopathological characteristics of the patients

**Fig. 4:**
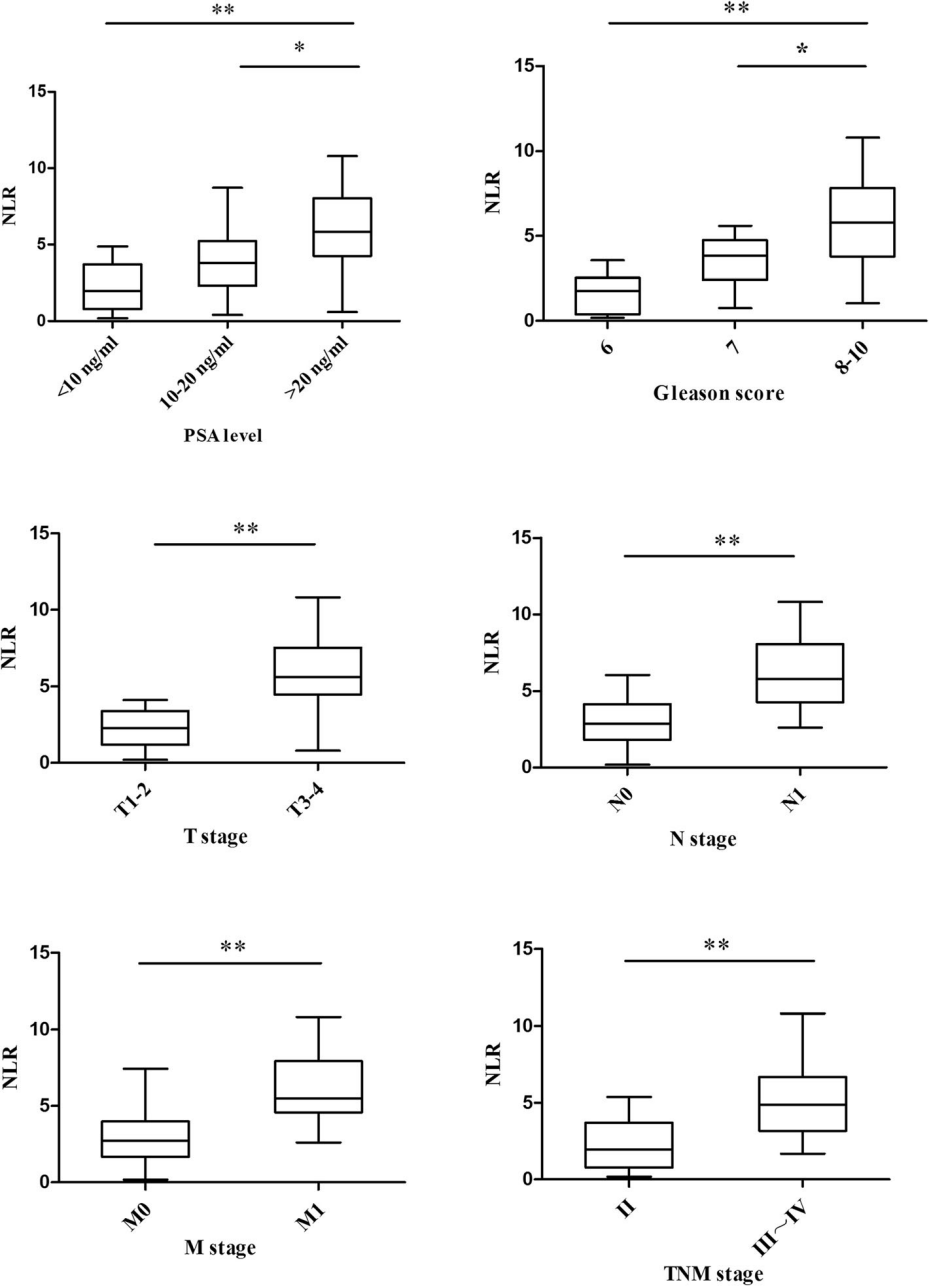
Box-plot graphics of NLR value in terms of PSA level, Gleason score, and TNM stage. Based on the standard definition, a plot represents median (horizontal line), the upper and lower lines of the box 75th and 25th. * *P*<0.05, ** *P*<0.01

According to the NLR cutoff value of 3.3, the 75 patients were divided into low NLR and high NLR groups (Table 3, Fig. 4). Among patients with PSA < 10 ng/mL, seven had NLR > 3.3, accounting for 36.8%. Among patients with PSA 10**–**20 ng/mL, 15 had NLR > 3.3, accounting for 51.7%, and among those with PSA > 20 ng/mL, 77.8% (*n* = 21) had NLR > 3.3. The χ^2^test showed that the difference was significant (χ^2^ = 8.248, *P* = 0.016). Among patients with a Gleason score ≤ 6, 7, and 8–10, 28.6% (*n* = 4), 57.1% (*n* = 16), and 69.7% (*n* = 13), respectively, belonged to the NLR high group (> 3.3). The difference was significant (χ^2^ = 6.797, *P* = 0.033). In addition, the percentage of patients with high NLR (> 3.3) in T3–4 stage patients was significantly higher than that in T1–2 stage patients (73.8% vs. 36.4%, χ^2^ = 10.593, *P* = 0.001). Patients with lymph node metastasis included a higher proportion of high NLR patients than those without lymph node metastasis (82.8% vs. 41.3%, χ^2^ = 12.495, *P* = 0.000), with similar results for distant metastasis (78.1% vs. 41.9%, χ^2^ = 9.863, *P* = 0.002). Similarly, in stage III-IV patients, there were more patients belonging to the NLR high group (> 3.3) than stage I-II patients (69.4% vs. 34.6%, χ^2^ = 8.396, *P* = 0.004). A higher pre-treatment NLR was associated with higher PSA level and Gleason score and a later clinical stage.

**Table 3:** Correlation between NLR value with clinicopathological features in prostate cancer

Patients and tumor characteristics	n(%)	NLR		χ2	*P*-value
		≤3.3	>3.3		
PSA level (ng/ml)					
< 10	19 (25.3)	12 (63.2)	7 (36.8)	8.248	0.016
10 ~ 20	29 (38.7)	14 (48.3)	15 (51.7)		
> 20	27 (36.0)	6 (22.2)	21 (77.8)		
Gleason score					
≤ 6	14 (18.7)	10 (71.4)	4 (28.6)	6.797	0.033
7	28 (37.3)	12 (42.9)	16 (57.1)		
8 ~ 10	33 (44.0)	10 (30.3)	23 (69.7)		
T stage					
T1–2	33 (44.0)	21 (63.6)	12 (36.4)	10.593	0.001
T3–4	42 (56.0)	11 (26.2)	31 (73.8)		
Lymph node metastasis					
N0	46 (61.3)	27 (58.7)	19 (41.3)	12.495	0.000
N1	29 (38.7)	5 (17.2)	24 (82.8)		
Distant metastasis					
M0	43 (57.3)	25 (58.1)	18 (41.9)	9.863	0.002
M1	32 (42.7)	7 (21.9)	25 (78.1)		
TNM stage					
Stage II	26 (34.7)	17 (65.4)	9 (34.6)	8.396	0.004
Stage III ~ IV	49 (65.3)	15 (30.6)	34 (69.4)		

*NLR* neutrophil-to-lymphocyte ratio, *PSA* prostate specific antigen

**Fig. 5:**
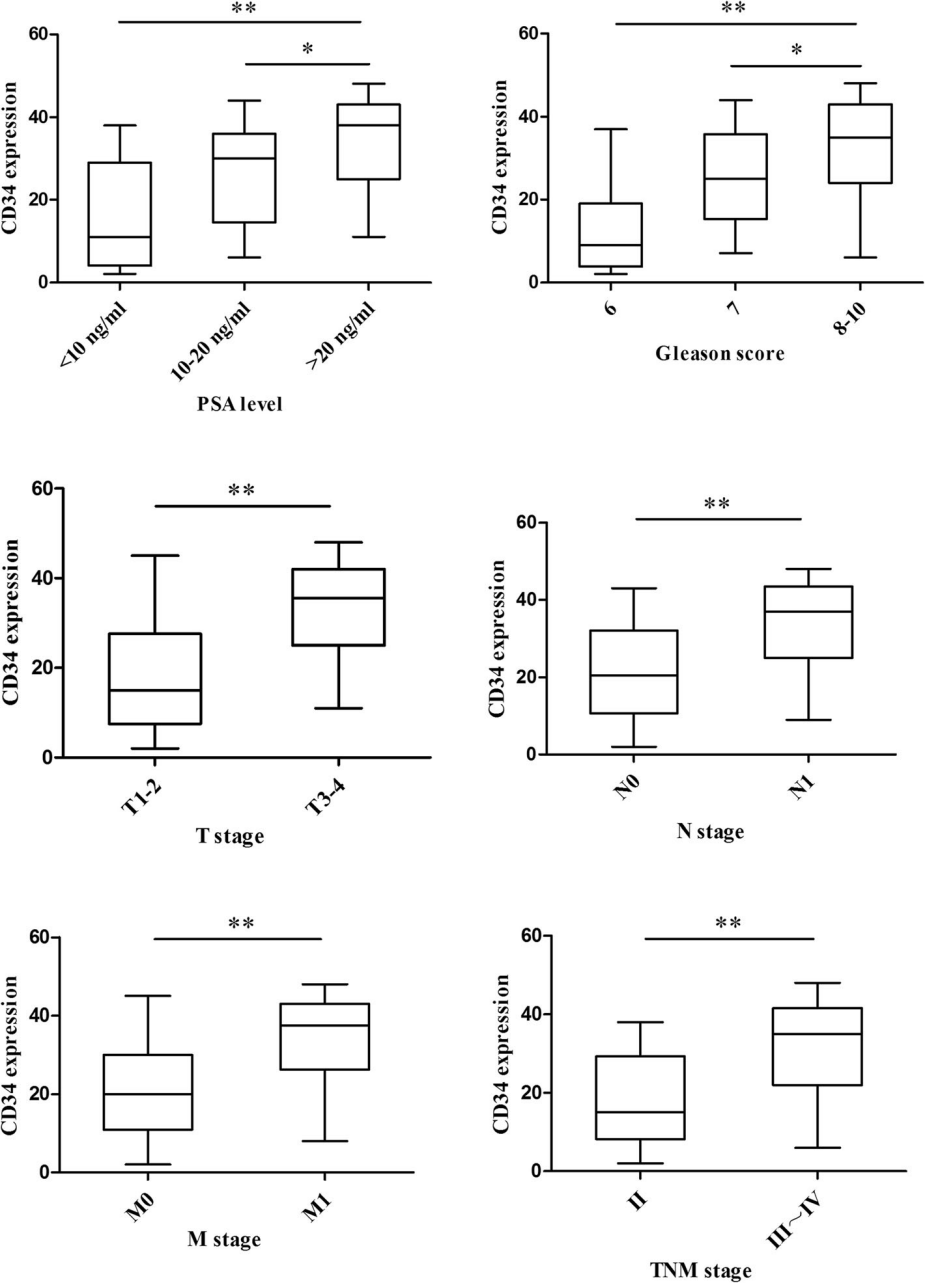
Box-plot graphics of CD34 expression in terms of PSA level, Gleason score, and TNM stage. Based on the standard definition, a plot represents median (horizontal line), the upper and lower lines of the box 75th and 25th. * *P*<0.05, ** *P*<0.01

Using 26 as the cutoff value, the 75 patients were divided into CD34 low-expression and high-expression groups (Table 4, Fig. 5). In patients with a PSA level < 10 ng/mL, 10–20 ng/mL, and > 20 ng/mL, the proportions of patients with high expression of CD34 were 26.3, 55.2, and 66.7%, respectively (χ^2^ = 7.465, *P* = 0.024). In patients with a Gleason score ≤ 6, 7, and 8–10, the proportions of patients with high expression of CD34 were 21.4, 46.4, and 69.7%, respectively (χ^2^ = 9.731, *P* = 0.008). The CD34 high-expression group included more stage T3–4 patients than stage T1–2 patients (71.4% vs. 27.3%, χ^2^ = 14.436, *P* = 0.000). Lymph node metastasis status was also correlated with CD34 expression. The proportion of patients with high expression of CD34 was significantly higher in patients with lymph node metastasis than in those without lymph metastasis (69.0% vs. 41.3%, χ^2^ = 5.452, *P* = 0.020). This pattern was also observed for distant metastasis, namely, patients with distant metastasis included a higher proportion of patients with CD34 high expression than those without distant metastasis (75.0% vs. 34.9%, χ^2^ = 11.829, *P* = 0.001). The proportion of CD34 high expression patients was significantly higher in stage III-IV patients than in stage II patients (63.3% vs. 30.8%, χ^2^ = 7.187, *P* = 0.007). Higher CD34 expression was associated with higher PSA level and Gleason score and later clinical stage.

**Table 4:** Correlation between CD34 expression with clinicopathological features in prostate cancer

Patients and tumor characteristics	n(%)	CD34		χ2	*P*-value
		≤26	>26		
PSA level (ng/ml)					
< 10	19 (25.3)	14 (73.7)	5 (26.3)	7.465	0.024
10 ~ 20	29 (38.7)	13 (44.8)	16 (55.2)		
> 20	27 (36.0)	9 (33.3)	18 (66.7)		
Gleason score					
≤ 6	14 (18.7)	11 (78.6)	3 (21.4)	9.731	0.008
7	28 (37.3)	15 (53.6)	13 (46.4)		
8 ~ 10	33 (44.0)	10 (48.0)	23 (69.7)		
T stage					
T1–2	33 (44.0)	24 (72.7)	9 (27.3)	14.436	0.000
T3–4	42 (56.0)	12 (28.6)	30 (71.4)		
Lymph node metastasis					
N0	46 (61.3)	27 (58.7)	19 (41.3)	5.452	0.020
N1	29 (38.7)	9 (31.0)	20 (69.0)		
Distant metastasis					
M0	43 (57.3)	28 (65.1)	15 (34.9)	11.829	0.001
M1	32 (42.7)	8 (25)	24 (75.0)		
TNM stage					
Stage II	26 (34.7)	18 (69.2)	8 (30.8)	7.187	0.007
Stage III ~ IV	49 (65.3)	18 (36.7)	31 (63.3)		

*PSA* prostate specific antigen

As shown in Table 5, patients with disease progression had significantly higher NLR and CD34 than those without disease progression (t = 3.865 and 4.392, *P* values were 0.011 and 0.000 for NLR and CD34).

**Table 5:** Correlation of NLR and CD34 with disease progression

	Without disease progression	Disease progression	t	*P*-value
NLR	2.37 ± 1.58	5.56 ± 2.19	3.865	0.011
CD34	21.48 ± 11.55	37.26 ± 10.71	4.392	0.000

*NLR* neutrophil-to-lymphocyte ratio

### Correlation between NLR/CD34 and clinicopathological characteristics of prostate cancer patients

The patients were divided into four groups based on NLR and CD34 values as follows: NLR^Low^/CD34^Low^, NLR^Low^/CD34^High^, NLR^High^/CD34^Low^, and NLR^High^/CD34^High^. ANOVA was used to analyze how the distribution of patients in these four groups varied according to PSA level, Gleason score, and clinical stage (Table 6). For PSA, 5 NLR^High^/CD34^High^ patients were in the PSA < 10 ng/mL group, accounting for 26.3%, and in the 10–20 ng/mL and > 20 ng/mL groups, there were 9 (31.0%) and 14 (51.9%) patients belonging to the NLR^High^/CD34^High^ group, respectively; the difference was significant (*P* = 0.011). Among patients with Gleason score ≤ 6, 7, and 8–10, there were 2, 7 and 19 patients belonging to the NLR^High^/CD34^High^ group, accounting for 14.3, 25.0, and 57.6%, respectively (*P* = 0.005). In patients with stage T3–4, lymph node metastasis, distant metastasis, and stage III-IV, there were 21 (50.0%), 19 (65.6%), 17 (53.1%), and 25 (51.0%) patients who were NLR^High^/CD34^High^, respectively (*P* < 0.05 for all). NLR^High^/CD34^High^ patients had higher PSA level and Gleason score and later clinical stage.

**Table 6:** Correlation between NLR/CD34 and clinicopathological features in prostate cancer

Patients and tumor characteristics	NLR/CD34				Standard Deviation	*P*-value
	NLR^Low^/CD34^Low^	NLR^Low^/CD34^High^	NLR^High^/CD34^Low^	NLR^High^/CD34^High^		
PSA level (ng/ml)						
< 10	8 (42.1)	1 (5.3)	5 (26.3)	5 (26.3)	0.169	0.011
10 ~ 20	10 (34.5)	6 (20.7)	4 (13.8)	9 (31.0)	0.142	
> 20	3 (11.1)	4 (14.8)	6 (22.2)	14 (51.9)	0.050	
Gleason score						
≤ 6	6 (42.9)	3 (21.4)	3 (21.4)	2 (14.3)	0.227	0.005
7	11 (39.3)	2 (7.1)	8 (28.6)	7 (25.0)	0.161	
8 ~ 10	4 (12.1)	6 (18.2)	4 (12.1)	19 (57.6)	0.059	
T stage						
T1–2	15 (45.5)	4 (12.1)	7 (21.2)	7 (21.2)	0.196	0.018
T3–4	6 (14.3)	7 (16.7)	8 (19.0)	21 (50.0)	0.084	
Lymph node metastasis						
N0	18 (39.1)	8 (17.4)	11 (23.9)	9 (19.6)	0.201	0.011
N1	3 (10.3)	3 (10.3)	4 (13.8)	19 (65.6)	0.173	
Distant metastasis						
M0	16 (37.2)	9 (20.9)	7 (16.3)	11 (25.6)	0.282	0.029
M1	5 (15.6)	2 (6.3)	8 (25.0)	17 (53.1)	0.186	
TNM stage						
Stage II	19 (73.1)	2 (7.7)	2 (7.7)	3 (11.5)	0.195	0.000
Stage III ~ IV	2 (4.1)	9 (18.4)	13 (26.5)	25 (51.0)	0.067	

*NLR* neutrophil-to-lymphocyte ratio, *PSA* prostate specific antigen

### Correlation between NLR and CD34

**Fig. 6:**
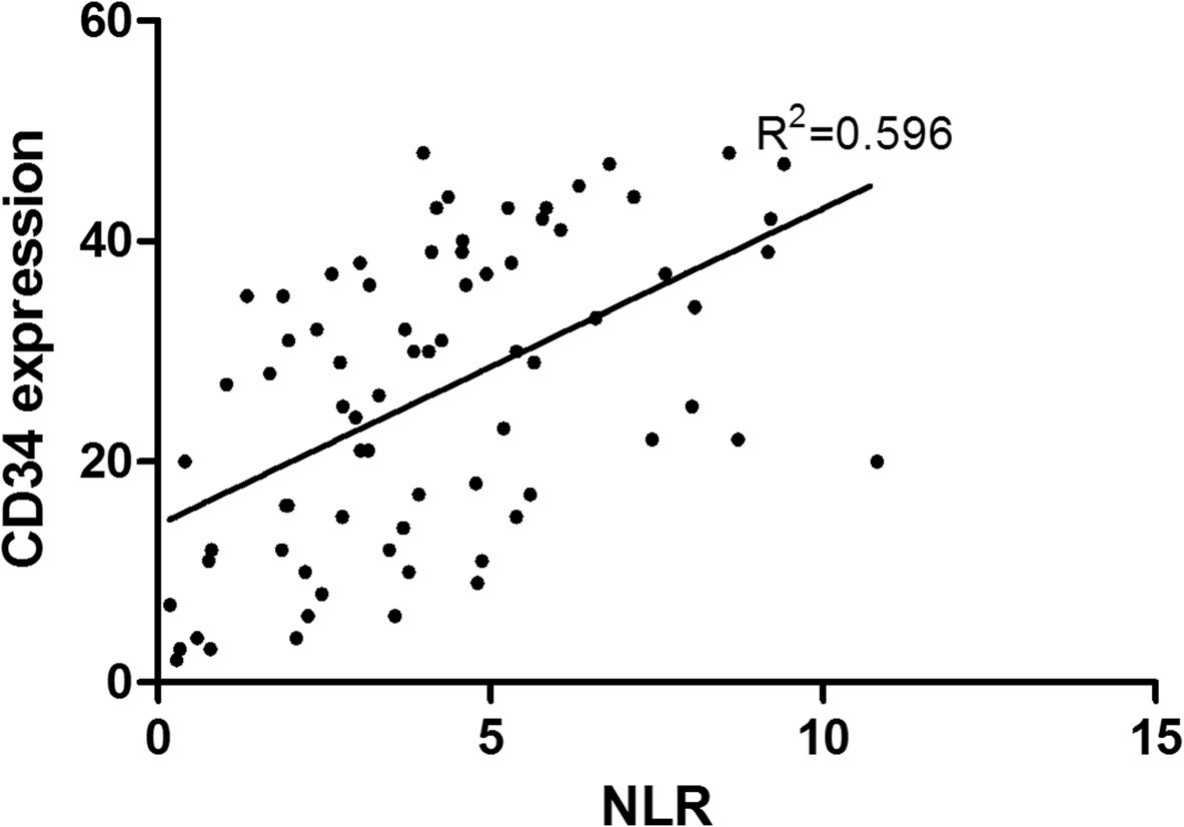
The correlation between the value of NLR and immunohistochemical expression of CD34 in prostate cancer patients. The value NLR showed a significant correlation with expression of CD34 (*r* = 0.529, *P* < 0.001)

The pre-treatment NLR and CD34 expression showed a positive correlation (*r* = 0.529, *P* < 0.001) by Pearson’s correlation analysis (Fig. 6).

### Progression-free survival (PFS) analysis

**Fig. 7:**
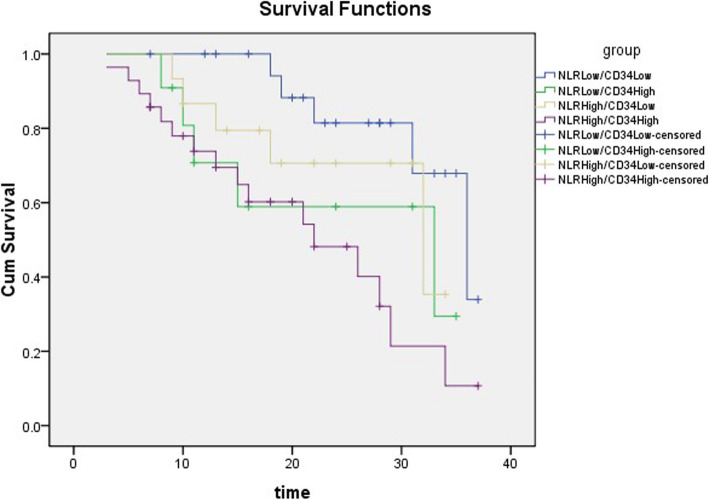
The effect of NLR and CD34 on disease-free survival after prostate cancer operation

The 75 patients were divided into four groups as follows: NLR^Low^/CD34^Low^, NLR^Low^/CD34^High^, NLR^High^/CD34^Low^, and NLR^High^/CD34^High^. Kaplan-Meier PFS estimation is shown in Fig. 7. The PFS of the patients in the NLR^High^/CD34^High^ group was significantly shorter than that in the NLR^Low^/CD34^Low^ group (*P* = 0.002, log-rank test).

## Discussion

The relationship between tumor development and inflammation has been widely studied. Oncogenesis involves interactions between the immune system, tumor cells, and tumor matrix cells. Chronic inflammation is a recognized risk factor for epithelial malignant tumors [13]. Inflammation can promote tumor development and progression, and tumor development may intensify inflammatory responses directly or through the microenvironment.

The present study suggested that NLR was a valuable marker for predicting the prognosis of prostate cancer. A higher pretreatment NLR was associated with higher PSA level, Gleason score, and later clinical stage. The exact mechanism underlying the association between increased NLR and poor prognosis of prostate cancer remains undefined; however, the following two aspects may be involved. On one hand, factors or a microenvironment that promote tumor growth also facilitate the production of neutrophils; the cytokines and inflammatory mediators produced by neutrophils promote the formation of the tumor microenvironment and stimulate the synthesis and release of vascular epithelial cell growth factor (VEGF), which strengthens tumor angiogenesis and progression [14, 15]. On the other hand, tumor cells, including prostate cancer cells, are abnormal cells that can induce a strong immune response in the body [16]. During this process, lymphocytes are consumed and the lymphocyte-mediated antitumor immune response is gradually weakened [17, 18].

Similar to NLR, a higher expression level of CD34 also predicted a higher PSA and Gleason score as well as a later clinical stage. This suggested a close association between CD34, an indicator reflecting tumor neovascularization activity, and the differentiation, stage, and prognosis of prostate cancer. CD34 is a single-pass transmembrane protein with a molecular weight of 105.120 kDa [19]. It is expressed on the surface of a variety of cells, particularly on vascular endothelial cells; therefore, CD34 is often used to label vascular endothelial cells [20, 21]. Moreover, CD34 is more likely to be expressed on newly-formed vascular endothelium [22]. A high expression of CD34 in tumor tissue indicates intensive tumor neovascularization and increased MVD, which was also shown in this study. An increase of MVD was associated with increased PSA and Gleason score and later clinical stage, which may be attributed to a rapid tumor growth induced by sufficient nutrient supply by newly-formed blood vessels.

The present results showed a positive correlation between pre-treatment NLR and CD34. This result provides evidence for the use of NLR in the evaluation of prognosis in prostate cancer. The increase of NLR suggested an activated immune response in the body. Prostate cancer cells themselves can produce proinflammatory cytokines to increase the production of neutrophils, which are involved in tumor progression via multiple pathways [23, 24]. Activated neutrophils can infiltrate tumor tissues and promote tumor metabolism by secreting a variety of bioactive molecules, such as VEGF and reactive oxygen species [14, 25]. The release of VEGF promotes tumor neovascularization and expression of CD34. Conversely, high expression of CD34 suggests increased tumor microangiogenesis. The physiological characteristics of tumor blood vessels are different from those of normal blood vessels. First, the permeability of the tumor vascular endothelium is greater than that of the normal vascular endothelium, making it easier for neutrophils to reach the tumor microenvironment. Second, tumor blood vessels are prone to necrosis and detachment, resulting in hypoxia in tumor tissues to form a hypoxic microenvironment, which induces tumor necrosis and an inflammatory response. Both factors increase the NLR.

## Conclusions

NLR and CD34 are mutually influential and causal. The present study provides experimental evidence for the potential of NLR in the prediction of prostate cancer prognosis. However, the study was limited by a small sample size and the single-center design, and further multi-center studies with a larger sample size are necessary to validate the present findings.

## Data Availability

The datasets used and/or analyzed during the current study are available from the corresponding author on reasonable request.

## Abbreviations

NLR: Neutrophil-lymphocyte ratio
PSA: Prostate specific antigen
MVD: Microvessel density
ROC: Receiver operator characteristic
ANOVA: Analysis of variance
VEGF: Vascular endothelial growth factor
AUC: Area under curve; CI: confidence interval
